# Towards Meaning in Life: A Path Analysis Exploring the Mediation of Career Adaptability in the Associations of Self-Esteem with Presence of Meaning and Search for Meaning

**DOI:** 10.3390/ijerph191911901

**Published:** 2022-09-21

**Authors:** Alessio Gori, Eleonora Topino, Andrea Svicher, Annamaria Di Fabio

**Affiliations:** 1 Department of Health Sciences, University of Florence, Via di San Salvi 12, Pad. 26, 50135 Firenze, Italy; 2 Department of Human Sciences, LUMSA University of Rome, Via Della Traspontina 21, 00193 Rome, Italy; 3 Department of Education, Languages, Intercultures, Literatures and Psychology (Psychology Section), University of Florence, 50135 Firenze, Italy

**Keywords:** well-being of workers, meaning in life, presence of meaning, search for meaning, career adaptability

## Abstract

In the contemporary world of work, workers are engaged more frequently in career choices to cope with changing work and working conditions. In this scenario, the well-being of workers is under threat. This study aims to examine the effect of career adaptability as a preventive resource on the relationship between self-esteem and meaning in life. Three-hundred Italian workers (67.3% females, mean age = 41.90; SD = 12.54) completed an online survey enclosing the Career Adapt-Abilities Scale, the Rosenberg self-esteem scale, and the Meaning in Life Questionnaire. Data were analyzed through a path analysis by implementing a mediation model to test the hypothesized relationship. The results show that career adaptability partially mediated the relationship between self-esteem and meaning in life, both considering the presence of meaning and the search for meaning dimensions. The findings expand current knowledge on the relationship between self-esteem, career adaptability and meaning in life in workers with implications for research and intervention.

## 1. Introduction

In the 21st century world of work, people are facing new challenges to managing the complexity of their careers and lives [1,2,3,4]. Nowadays, workers have to cope with more frequent career transitions, experiencing higher flexibility and insecurity in working life [5,6], which also has negative effects on their emotional and relational well-being [7,8,9,10,11]. Furthermore, organizational environments are becoming more fluid and unstable, delegating the development of career paths to workers [12,13]. As a consequence, workers are engaged more frequently in career choices than in the past [14]. In this new reality, the well-being of workers is threatened [1]. To help workers cope with this scenario, career counseling research has shifted the emphasis from a career development point of view to a career management perspective [2,4] for intervention. In this framework, the overlap between working and other dimensions of the worker’s life are considered inseparable [2,4,15]. Thus, from the career counseling domain emerged a changing approach, aiming to enable workers to manage their careers and their lives, rather than to only make work-related decisions [14,16,17,18], going further as preventive actions for workers in the workplace [19,20]. The mainstay of this perspective is to help workers construct meaningful personal and professional projects, defining who and what they want to become in work and across their lives [16]. At the same time, helping workers to move their career/life project from a motivational to a meaning paradigm [21,22] supports them in discovering and enhancing intrinsic energy.

Career counselling research has extensively investigated the association between career and meaning-related variables (e.g., [23,24,25,26,27,28,29]). In line with this, most studies on adult working people refer to concepts of meaning associated with working life, such as meaningful work and calling (e.g., [23,25,27]). Differently, less of the literature describes the association between career-related variables and concepts of meaning expanded to whole life domains, even though contemporary career perspectives consider life and career domains to be entwined [15]. Moreover, research on careers and meaning in life mainly focused on samples of college students [24,26,28,29,30,31] and older workers [32,33], showing only a handful of studies conducted on adult working people [34,35,36,37,38]. The same gap is valid for research addressing relationships between career adaptability and meaning in life [39]. It is focused on those who traditionally transition within their careers (students/older workers) and not on working people, even though career transitions in the contemporary world of work are extended to the entire course of working life [1,2,3,4]. Lastly, the literature has established that self-esteem is an individual variable deeply involved in career construction [40,41,42]. However, the relationship between career adaptability and meaning in life is, to our knowledge, unexplored in workers. Thus, to overcome these gaps, the current study examines the relationship between self-esteem, career adaptability and meaning in life, focusing on working adults.

### 1.1. Meaning in Life

Meaningfulness is commonly considered as a positive variable in eudaimonic theories of well-being [43,44] and an indicator of eudaimonic well-being itself [45]. It has been directly equated with authentic living [46], and strictly associated with a set of growth-related variables that are thought to provide the conditions from which growth and psychological strengths arise [19,21]. In line with this, the concept of meaning in life has gained increasing attention in career counseling and vocational behavioral science [23,47]. It was introduced by Viktor Frankl [48] to help clients explore the meaning associated with life’s experiences. Subsequently, Super, Savickas, and Super [49] conceived meaning in life as a facilitator also capable of helping clients to identify the role of values in promoting career adaptation and decision-making processes. More recently, Steger et al. [50] advanced a comprehensive definition of perceived meaning in life as “the sense made of, and significance felt regarding, the nature of one’s being and existence” (pp. 81). Moreover, they differentiated between the presence of meaning (the awareness that one’s life is meaningful) and the search for meaning (the drive and orientation toward finding meaning in one’s life) [50]. The presence of meaning was found to be associated with a number of positive variables, such as well-being, as well as personality and religiosity variables [50]. Differently, the search for meaning was labelled as a variable also reflective of the frustrations associated with the search for meaning in life that may be distressing [50]. The search for meaning showed medium correlations with negative effects, neuroticism [50], depression, and negative career thoughts [31]. The literature showed that meaning in life was associated with a favorable development of career of individuals during their lifespan [33,35,36,51]. With regards to workers, meaning in life was found to be linked with job performance [37], job satisfaction [52], organizational commitment, and organizational citizenship behaviors [36]. Other results from social workers illustrated that subjective well-being and compassion satisfaction (as the satisfaction that comes from being able to help others through one’s caring work) were positively associated with meaning in life [35]. Similarly, data from older workers highlighted that meaning in life was highly correlated with overall job satisfaction [33]. 

### 1.2. Career Adaptability

In the rapidly changing occupational landscape, the need for flexibility, resilience, and adaptability in constructing individuals’ careers is another compelling challenge [53]. During the last three decades, scholars have begun to study career adaptability as a pillar to the identification of crucial components in enhancing clients’ career readiness [54,55,56]. Career adaptability arises from the concept of career maturity, which is the centerpiece of Super’s career development theory [57], and it has been updated by scholars during the last three decades [58,59]. Super and Knasel [59] advanced the idea to shift “career maturity” into “career adaptability”. In this view, career adaptability refers to the individual’s capacity to cope with the transition of their career roles, finding a balance between career roles and the pressure from work environments [59]. Savickas [58,60,61] expanded upon the concept of career adaptability and defended it as an individual’s readiness and psychosocial resources to cope with changing work and working conditions (e.g., occupational transitions, developmental vocational tasks, or work traumas). Career adaptability involves the ability of clients to adapt to changing tasks, remain engaged in continued self-learning, and regulate their career direction [58]. Savickas conceived career adaptability to be composed of four main psychosocial strengths or capacities: concern, control, curiosity, and confidence [58]. Concern deals with the client’s future work life. Control encompasses the client’s career future. Curiosity encloses the exploration of possibilities for the client’s future selves and scenarios. Confidence is related to the seeking of one’s career goals [58,60,61]. A literature review [62] and a meta-analysis [63] showed that career adaptability was correlated with adaptivity variables (i.e., cognitive ability, big five traits, self-esteem, core self-evaluation, proactive personality, future orientation, hope, and optimism), adapting response (career planning, career exploration, and self-efficacy), and adaptation results (career identity, career calling, job/career/school satisfaction, job stress, employability, promotability, work performance, turnover intentions, income, engagement, and subjective well-being). More particularly, meta-analytic findings showed that career adaptability partially mediated the relationship between adaptivity variables and adaptation results [63].

### 1.3. Self-Esteem

Self-esteem is an individual variable that was found to be deeply involved in the career construction processes [40,41,42]. Self-esteem is the positive evaluation of individuals about themselves as persons [64]. Individuals with high self-esteem are more prone to see themselves as worthy, capable, and significant, whereas those with low self-esteem are more inclined to doubt their abilities and self-worth [65]. In line with the self-verification theory [66], individuals need to have a strong coherence between their self-view and the activities in which they are engaged. Individuals with high self-esteem are those with high self-image, self-perceived competence, and success expectancy. Thus, in turn, a high level of self-esteem could drive individuals to be set and engaged in challenging career goals, as well as to verify and confirm their positive self-image [42]. Moreover, self-esteem was found to be associated with career adaptability. Meta-analytic results identified self-esteem among those variables that are associated with adaptivity (i.e., the extent to which people are willing to meet the vocational tasks with fitting responses). In the career construction model of adaptation [61,67], adaptivity positively influences career adaptability which, in turn, positively influences adaptation results. Several findings showed that self-esteem was found to be strongly associated with career adaptability [40,41,42] and self-esteem predicted career adaptability [42]. Only a study run on students showed that self-esteem does not have an effect on students’ career adaptability [68].

### 1.4. Purpose of the Study

With this in mind, the aim of the present study is to investigate the relationship between self-esteem, career adaptability, and meaning in life. According to the career construction model of adaptation [61,63,67], self-esteem is an adaptivity variable. In line with this model, meta-analytic results showed that self-esteem and career adaptability were positively and strongly associated, and longitudinal findings showed that self-esteem predicted career adaptability [63]. To the best of our knowledge, only one previous study examined the association between career adaptability and meaning in life, showing a positive association between the two variables [30]. Only a handful of studies showed data on the relationship between self-esteem and meaning in life. Barnett et al. [34] showed that self-esteem mediated the relationship between the presence of meaning in life and psychological distress; Zhang et al. [32] and Du et al. [38] found that meaning in life and self-esteem were associated. However, to the best of our knowledge no previous study has investigated the association between self-esteem, career adaptability, and meaning in life, while also taking into account the difference between the presence of meaning and the search for meaning. According to the career construction model of adaptation [61,63,67] and the meaning paradigm [22], career adaptability could mediate the relationship between self-esteem and meaning in life. In this view, the strengths enclosed in career adaptability (concern, control, curiosity, and confidence) could facilitate eudaimonic well-being in terms of meaning in life. Concerning the mediation effect of career adaptability, on the basis of previous findings [30,32], we hypothesize a partial mediation. Thus, on the basis of the literature the following hypotheses are tested:

**Hypothesis** **1** **(H1).** *Self-esteem will be positively associated with career adaptability.*

**Hypothesis** **2** **(H2).** *Self-esteem will be positively associated with the presence of meaning*.

**Hypothesis** **3** **(H3).** *Self-esteem will be negatively associated with the search for meaning*.

**Hypothesis** **4** **(H4).** *Career adaptability will be positively associated with the presence of meaning*.

**Hypothesis** **5** **(H5).** *Career adaptability will be negatively associated with the search for meaning*.

**Hypothesis** **6** **(H6).** *Career adaptability will partially mediate the relationship between self-esteem and the presence of meaning*.

**Hypothesis** **7** **(H7).** *Career adaptability will partially mediate the relationship between self-esteem and the search for meaning*.

## 2. Method

### 2.1. Participants and Procedure

The sample involved in this research consisted of 300 Italian workers (67% women, 33% men), with an age ranging from 18 to 69 years (*M* = 41.90; *SD* = 12.54). As shown in Table 1, they were predominantly single (41%) or married (37%). Concerning their educational and professional condition, most of them declared they had a high school diploma (40%) or a master’s degree (31%), and they claimed to work as an employee (75%). Participants were recruited online through a snowball method, starting with a call for participants posted on various social media. The snowball sampling is a nonprobability sampling technique, within which primary selected sources of data are also asked to disseminate the survey among their contacts [69]. The survey was hosted on the Google Forms platform and the survey was launched from 20 January 2022 to 30 January 2022. Each participant replied to the self-report questionnaires after being informed about the general objective of the research and electronically providing informed consent. Participation was voluntary and confidentiality was assured. All participants provided written and informed consent according to Italian privacy laws (Law Decree DL-196/2003) and European Union General Data Protection Regulation (EU 2016/679).

### 2.2. Measures

#### 2.2.1. Meaning in Life Questionnaire (MLQ)

The *Meaning in Life Questionnaire* (MLQ [50]; Italian version [70]) is a 10-item self-report scale used to assess the constructs of the presence of meaning (e.g., “*I understand my life’s meaning*.”) and of the search for meaning (e.g., “*I am searching for meaning in my life*.”). Each dimension was assessed by five items, rated on a five-point Likert scale ranging from 1 (absolutely untrue) to 7 (absolutely true). Both subdimensions were obtained by summing five respective items. The greater the scores were, the higher the levels of presence of meaning and search for meaning. The Italian version used in this research showed good internal consistency, with Cronbach’s coefficients of 0.87 for both the assessed dimensions.

#### 2.2.2. Rosenberg Self-Esteem Scale (RSES)

The *Rosenberg self-esteem scale* (RSES [71]; Italian version [72]) is a 10-item self-report scale used to assess global self-esteem. Items were rated on a four-point Likert scale, ranging from 0 (strongly agree) to 3 (strongly disagree). The total score was obtained by summing all the items. The greater the score was, the higher the self-esteem. The Italian version used in this research showed excellent internal consistency in the present sample, with a Cronbach’s coefficient of 0.90.

#### 2.2.3. Career Adapt-Abilities Scale (CAAS)

The *Career Adapt-Abilities Scale* (CAAS [58]; Italian version [73]) is a 24-item self-report scale used to assess the levels of the respondent’s career adaptability. Items were rated on a five-point Likert scale, ranging from 1 (not a strength) to 5 (greatest strength), and could be grouped into the dimensions of concern, control, curiosity, and confidence. A total score was obtained by calculating the mean response across all 24 items. The greater the score was, the higher the self-esteem. The Italian version used in this research showed excellent internal consistency in the present sample, with a Cronbach’s coefficient of 0.95.

### 2.3. Data Analysis

Data were computed using the Statistical Package for Social Sciences version 21 (IBM SPSS Statistics 21, SPSS Inc., Chicago, IL, USA) and AMOS statistical package version 24.0 [74]. First, Pearson’s *r* correlation analysis was implemented to explore the associations between all the included variables. Then, path analysis modeling was performed, where significance was indicated by *p* values under 0.05 for both direct and indirect effects. Model fit statistics included: the Chi-square model (χ^2^; *p* > 0.05 indicative of good fit [75]); the non-normed fit index (NNFI; 0.90 or above indicative of good fit [76]); the comparative fit index (CFI; 0.95 or above indicative of good fit [76]); the root-mean-square error of approximation (RMSEA; 0.08 or below indicative of reasonable fit [77]); the standardized root-mean-square residual (SRMR; 0.08 or below indicative of good fit [78]). Finally, the statistical stability of the model was tested through the bootstrapping method with 95% bias-corrected confidence intervals and 5000 bootstrapped samples.

## 3. Results

As shown in Table 2, the presence of meaning was significantly and positively associated with career adaptability (*r* = 0.508, *p* < 0.01), and self-esteem (*r* = 0.615, *p* < 0.01), which were in turn significantly and positively related to each other (*r* = 0.546, *p* < 0.01). Differently, the search for meaning showed significant and negative correlations with the presence of meaning (*r* = −0.350, *p* < 0.01), career adaptability (*r* = −0.349, *p* < 0.01), and self-esteem (*r* = −0.409, *p* < 0.01).

**Figure 1:**
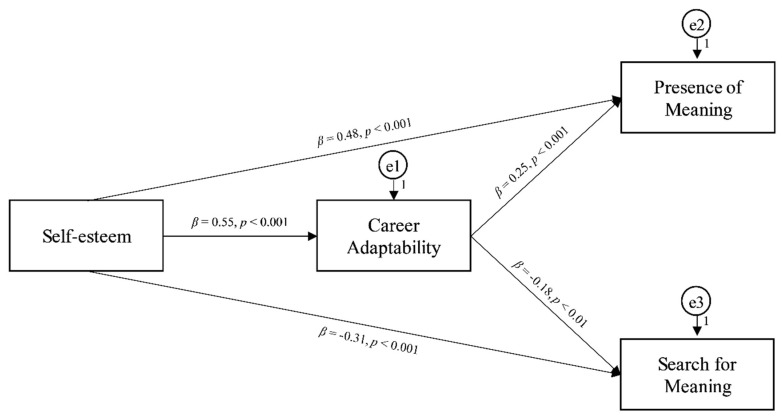
Path analysis model: the mediation of career adaptability in the associations of self-esteem with presence of meaning and search for meaning.

Concerning the path analysis, the proposed mediation model showed a good model fit: *χ*^2^ (1) = 2.861 (*p =* 0.091), NNFI = 0.966, CFI = 0.004, RMSEA = 0.079, SRMR = 0.021 (see Figure 1).

Specifically, self-esteem was significantly and positively associated with career adaptability (*β* = 0.55, *p* < 0.001) (H1 was confirmed), the mediating variable. Furthermore, significant total effects were found in the positive relationships of self-esteem with the presence of meaning (*β* = 0.62, *p* < 0.001) (H2 was confirmed) and the negative relationships with the search for meaning (*β* = −0.41, *p* < 0.001) (H3 was confirmed). In turn, career adaptability was significantly and positively related to the presence of meaning (*β* = 0.25, *p* < 0.001), (H4 was confirmed) and significantly and negatively associated with the search for meaning (*β* = −0.18, *p* < 0.001) (H5 was confirmed). Inserting career adaptability as the mediating variable, career adaptability mediated the positive relationship between self-esteem and the presence of meaning, although this relationship remained significant (*β* = 0.48, *p* < 0.001), suggesting a partial mediation (H6 was confirmed). Furthermore, career adaptability also mediated the negative relationship between self-esteem and the search for meaning, although this relationship remained significant (*β* = −0.31, *p* < 0.001), suggesting a partial mediation (H7 was confirmed). Finally, the bootstrapping method confirmed the statistical stability of the model (see Table 3).

## 4. Discussion

In the midst of all the challenges that are taking place in the 21st-century scenario, career counseling aims to promote new paths to enable workers to effectively deal with the changing world of work and the working conditions framework [1,14,16,17,18]. This has required career researchers to discover new strategies to help clients to cope with such a challenging new reality [5]. The literature showed that the career construction model of adaptation [61,63,67] and the shift from a motivational to meaning paradigm [22] are promising frameworks in strengthening the readiness of workers. Starting from these premises, the current research aimed to better understand the factors that may influence the meaning in life as a valuable source in discovering the intrinsic energy to cope with career and life challenges [22,79,80]. In particular, this study aimed to deepen the link between self-esteem and meaning in life by also considering the effects of career adaptability.

The results show a significant and positive link between self-esteem and career adaptability (H1 was confirmed). This is in accordance with past findings, highlighting a positive association between self-esteem and career adaptability (e.g., [63]). Furthermore, it is in line with the career construction model of adaptation [61,63,67] that considers self-esteem as an adaptivity variable in relation to career adaptability.

The findings also reveal that self-esteem was found to be associated with the presence of meaning and the search for meaning, in line with previous findings [32]. However, our results build upon previous findings which showed that self-esteem was positively associated with the presence of meaning (H2 was confirmed), whereas self-esteem was negatively associated with the search for meaning (H3 was confirmed). To the best of our knowledge, our study is the first study that investigates the relationship between self-esteem and meaning in life as conceived by Steger et al. in workers [50]. Thus, we cannot compare the present results with the literature. However, the findings can be explained by considering the self-verification theory [41]: individuals with high self-esteem are those engaged in goals to verify and confirm their positive self-image. According to Steger et al. [50], the presence of meaning is the awareness that one’s life is meaningful. Thus, it could be possible that individuals with high self-esteem are also those with a higher presence of meaning since this link could serve as a reinforcement of their positive self-image. Differently, self-esteem was found to be negatively associated with the search for meaning. This could be explained by the fact that the search for meaning is also reflective of frustration [50]. Thus, the negative association between self-esteem and the search for meaning could be explained by the fact that this latter variable may not serve as reinforcement of a positive self-image.

Career adaptability was found to be positively associated with the presence of meaning (H4 was confirmed), in line with previous findings [44]. This positive link could be explained according to the career construction model of adaptation [61,63,67], in which career adaptability is positively related to *adaptation results*, including variables concerning a positive reappraisal of career path (e.g., career calling, well-being, and career identity). Thus, the presence of meaning could be hypostatized as an *adaptation result* of career adaptability. Differently, career adaptability was found to be negatively associated with the search for meaning (H5 was confirmed). This association could be explained by considering career adaptability itself [58]. It encompasses the feature of remaining engaged in continued self-learning, adaptively regulating the career direction [58]. On the contrary, the search for meaning deals with the frustration. Thus, career adaptability, maintaining workers in active engagement and self-learning could be useful in relation to the search for meaning.

Lastly, our results show that career adaptability partially mediated the positive association between self-esteem and the presence of meaning (H6 was confirmed), as well as the negative association between self-esteem and the search for meaning. This is consistent with the career construction model of adaptation [61,63,67] and the meaning paradigm [22]. In line with this, career adaptability may operate as a process that maximizes the positive association between self-esteem and the presence of meaning as an *adaptation result* [61,63,67]. On the other hand, career adaptability may also reduce the negative association between self-esteem and the presence of meaning, ameliorating the feeling of frustration and helping workers to remain engaged in continued self-learning [61,67].

These results could also open promising lines of research in the field of career study that want to help workers to construct meaningful personal and professional projects [1,16]. In this framework, career adaptability could be part of the positive psychological resources and strengths to be promoted in workers to enhance the shift from a motivational to a meaning paradigm [22].

These findings should be interpreted with caution due to some limits that should be addressed. First, data were collected online through a snowball procedure (nonprobability sampling technique), in which an anonymous link was spread: this could have excluded a portion of the population (e.g., those who did not have internet access); therefore, the participants recruited in this study may not be representative of the general population. Furthermore, only self-report measures were used to collect the data, by consequently assessing several factors for each participant: this exposes the results to the risk of several biases (e.g., social desirability, common method bias), which could limit the veracity of the information obtained. Future research could overcome these limitations by integrating different methods of recruiting and data collection, (e.g., also using structured interviews in a one-to-one setting) and by adding a time delay during the administration. Moreover, no information was collected concerning the geographic provenience of the respondents, nor the specific job area of workers. Future research could replicate and expand upon our results by analyzing the influence of these factors on the relationships between the variables assessed in this research, comparing the specific worker populations and using survey-weighted estimations to increase the accuracy in the generalizability of the data. Additionally, this research implemented a cross-sectional design that impedes the clear establishment of causal inferences in the relationship between the variables involved in the applied model. In future research, a longitudinal approach could help to give further evidence, deepening and extending these results. Future research could integrate and expand the given findings by implementing longitudinal data and assessing vulnerable workers that are in need of more carefully assistance in their career–life projects [81,82,83].

## 5. Conclusions

In brief, the findings of this study highlighted the relationship between self-esteem, career adaptability, and meaning in life. More specifically, the results of this research showed that the levels of self-esteem in workers were positively associated with the presence of meaning and negatively associated with the search for meaning, both with a direct and indirect path. In the indirect path, specifically, self-esteem showed a significant role in favoring the presence of meaning through its positive effect on career adaptability on the one hand, which in turn had a positive association with the presence of meaning, increasing the perception of having a precise and satisfactory direction in one’s life; on the other hand, self-esteem showed a positive effect on career adaptability, which in turn has a negative relationship with the search for meaning, reducing the feeling of frustration linked to the search for meaning. This may have interesting practical implications in enriching interventions to favor the shift from a motivational to meaning paradigm in workers [22], suggesting the relevance of increasing psychological resources to career adaptability. Furthermore, career adaptability is confirmed to be a fundamental variable in career counseling approaches, as well as in preventive interventions at the workplace to provide workers with meaningful careers and lives [14,17,18,22,84] for decent work and decent lives [85,86].

## Figures and Tables

**Table 1:** Demographic and professional characteristics of the sample (*N* = 300).

Characteristics		M ± SD	N (*%*)
Age		41.90 ± 12.54	
Sex			
	*Males*		98 (32.7%)
	*Females*		201 (67.3%)
Marital Status			
	*Single*		124 (41.3%)
	*Married*		110 (36.7%)
	*Cohabiting*		35 (11.7%)
	*Separated*		10 (3.3%)
	*Divorced*		15 (5.0%)
	*Widowed*		6 (2.0%)
Education			
	*Elementary School diploma*		1 (0.3%)
	*Middle School diploma*		18 (6.0%)
	*High School diploma*		121 (40.3%)
	*University degree*		34 (11.3%)
	*Master’s degree*		91 (30.7%)
	*Post-lauream specialization*		34 (11.3%)
Occupation			
	*Employee*		225 (75.0%)
	*Freelance*		33 (11.0%)
	*Entrepreneur*		19 (6.3%)
	*Trader*		5 (1.7%)
	*Artisan*		8 (2.7%)
	*Manager*		10 (3.3%)

**Table 2:** Correlation matrix.

	Search for Meaning	Presence of Meaning	Career Adaptability	Self-Esteem
Search for Meaning	1	−0.350 **	−0.349 **	−0.409 **
Presence of Meaning		1	0.508 **	0.615 **
Career Adaptability			1	0.546 **
Self-Esteem				1

Note: Bold values indicate significant *p*-values. **. Correlation is significant at the 0.01 level (2-tailed).

**Table 3:** Coefficients of the mediation model.

Effect	Estimate	SE	*p*	BootLLCI	BootULCI
*Total effects*					
Self-Esteem → Presence of Meaning	0.590	0.047	<0.001	0.497	0.681
Self-Esteem → Search for Meaning	−0.468	0.058	<0.001	−0.581	−0.351
*Direct effects*					
Self-Esteem → Presence of Meaning	0.461	0.050	<0.001	0.353	0.570
Self-Esteem → Search for Meaning	−0.356	0.071	<0.001	−0.474	−0.224
*Indirect effects*					
Self-Esteem → Presence of Meaning	0.128	0.032	<0.001	0.072	0.199
Self-Esteem → Search for Meaning	−0.112	0.038	0.001	−0.190	−0.042
*Mediation paths*					
Self-Esteem → Career Adaptability	1.374	0.122	<0.001	10.115	10.639
Career Adaptability → Presence of Meaning	0.093	0.020	<0.001	0.054	0.133
Career Adaptability → Search for Meaning	−0.082	0.028	0.004	−0.137	−0.030

## Data Availability

The data presented in this study are available on request from the corresponding author on reasonable request. The data are not publicly available due to privacy reasons.
